# Comparative Study in Quality of Life between Thai Endometrial Cancer Survivors and Healthy Women in Thammasat University Hospital

**DOI:** 10.31557/APJCP.2020.21.1.249

**Published:** 2020

**Authors:** Sarunya Sivapornpan, Komsun Suwannarurk, Kankamol Jaisin, Junya Pattaraarchachai, Kornkarn Bhamarapravatana

**Affiliations:** 1 *Department of Obstetrics and Gynecology, *; 2 *Gynecologic Oncology Unit, Department of Obstetrics and Gynecology, *; 3 *Department of Psychiatry, Faculty of Medicine, *; 6 *Department of Preclinical Science, Thammasat University, Pathumthani, *; 4 *Department of Psychiatry, Faculty of Medicine Siriraj Hospital, Mahidol University, *; 5 *Chulabhorn International College of Medicine, Bangkok, Thailand. *

**Keywords:** Endometrial cancer, quality of life, cancer survivors

## Abstract

**Background:**

This study aimed to survey quality of life (QoL) in endometrial cancer survivors between surgery with adjuvant therapy (radiation with or without chemotherapy) and surgery alone in Thammasat University Hospital, Thailand.

**Materials and Methods:**

This cross-sectional study was conducted at the Gynecologic Oncology clinic, Thammasat University hospital, Thailand between March 2011 and May 2019. Participants were endometrial cancer cases who underwent surgical staging with or without adjuvant treatment (study) and healthy women who came to gynecologic department for annual cervical screening (control). Assessment of QoL was investigated via the structural questionnaire designed by the European Organization for Research and Treatment of Cancer (EORTC) QLQ-C30 (Thai version).

**Results:**

During the period of the study, 94 participants who were diagnosed with endometrial cancer and underwent surgical staging were enrolled. There were 51, 43 and 51 cases in group A (surgery with adjuvant therapy), group B (surgery only) and group C (control), respectively. Control cases were participants who had comparable demographic characteristics and underwent gynecological checkup during the period of study. In part of physical functioning, group B had statistically better scores than group A. Participant in group B and C reported significantly better QoL in part of social functioning than group A. Symptom severity; appetite loss and constipation in group B was statistically less than in group A. Constipation problems in group A and C were comparable. Participants in group C had worse global health status than group A/B.

**Conclusion:**

Adjuvant treatment with either radiation or chemotherapy had negative impacts on QoL in endometrial cancer survivors. It impacted physical health, social function, appetite loss, and constipation. All endometrial cancer survivors had global health scores better than healthy peers. Thoroughly counseling to endometrial cancer survivors remains an important tool for increasing awareness of treatment complications and lowering psychological emotional stress.

## Introduction

Endometrial cancer is a gynecologic malignancy that occurs in women most commonly in their sixth and seventh decade. Most cases had underlying diseases i.e., diabetes mellitus (DM), hypertension (HT) and dyslipidemia (DLP). It is a hormone dependent cancer with either unopposed estrogen condition or exogenous stimulation from tamoxifen (Dowdy et al., 2019).

Endometrial cancer is the 6^th^ most commonly occurring cancer in women (Bray et al., 2018). In Thailand, endometrial cancer is the 9th most common form of female cancer with age standardized incidence rate of 4.3/100,000 women per year (Wilailak et al., 2016).

Most endometrial cancer was diagnosed at an early stage. Based on the National Comprehensive Cancer Network (NCCN) guideline, the primary treatment is surgical staging including hysterectomy, bilateral salpingo-oophorectomy, pelvic and/or paraaortic lymph node biopsy (Dowdy et al., 2019). Adjuvant treatment including vaginal brachytherapy, chemotherapy and concurrent chemotherapy (CCRT) were performed according to the histopathology report and staging (Dowdy et al., 2019).

Endometrial cancer survivors (ECS) were subjected to surgical intervention, radiation and chemotherapy. Adjuvant treatment after surgery of chemotherapy and/or radiation produced morbidity due to surrounding normal tissue injury (Dowdy et al., 2019). Atrophic vagina, urinary bladder, as well as small and large bowel complications compromised quality of life (QoL) in these cancer survivors.

The objective in this study was to survey the quality of life in endometrial cancer survivors between surgery with adjuvant therapy (radiation with or without chemotherapy) and surgery alone.

## Materials and Methods

This study was approved by the human research ethics committee of Thammasat University (MTU-EC-OB-2-200/61). This cross-sectional study was conducted at Thammasat University hospital, Thailand between March 2011 and May 2019. Participants were endometrial cancer cases who underwent surgical staging with or without adjuvant treatment (study group) and healthy women who came to gynecologic department for annual cervical screening (control group). QoL of these cases was surveyed by the structural questionnaire with a single physician. Tools for assessment of QoL was the questionnaire designed by the European Organization for Research and Treatment of Cancer (EORTC) QLQ-C30 (Thai version) (Aaronson et al., 1993). Inclusion criteria included participants who were between 30-70 years old and were able to complete the questionnaire in Thai language. Exclusion criteria included refusal or inability to communicate in Thai language.

The study’s questionnaire comprised of 5 functional scales, 9 symptom scales and global health status. It was used to evaluate the quality of life in cancer patients. Functional scales consisted of physical functioning (FPF), role functioning (FRF), emotional functioning (FEF), cognitive functioning (FCF) and social functioning (FSF). Symptom scales consisted of fatigue, nausea/vomiting, pain, dyspnea, insomnia, appetite loss, constipation, diarrhea and financial difficulties. Global health status scales were expressed by the patient’s evaluation of their wellbeing on a scale of one to seven. Higher scores of functioning scales and global health status indicated higher quality of life while the lower scores of symptom scales indicated positive outcomes (less unpleasant symptoms).

The participants’ demographic characteristics in this study comprised of age, body mass index (BMI), occupation, income, education level, religion, marital status, social support, health insurance, history of underlying disease/other surgery, parity and family history of cancer. Cancer information consisted of manner of treatment, endometrial cancer stage, histology type and grading.

Statistical analysis was evaluated by using commercial statistic software (Statistical Package for the Social Science for window version 23). Continuous data was expressed by mean and standard deviations. Independent T test was used for category data evaluation. Quality of life score in the present study did not conform to a normal distribution. Non parametric methods by Kruskal-Wallis H were chosen for statistical evaluation. Multiple comparisons of QoL among the study and control groups were performed by Conover’s test. The statistical significance was set at *p*-value less than 0.05.

## Results

**Figure 1:**
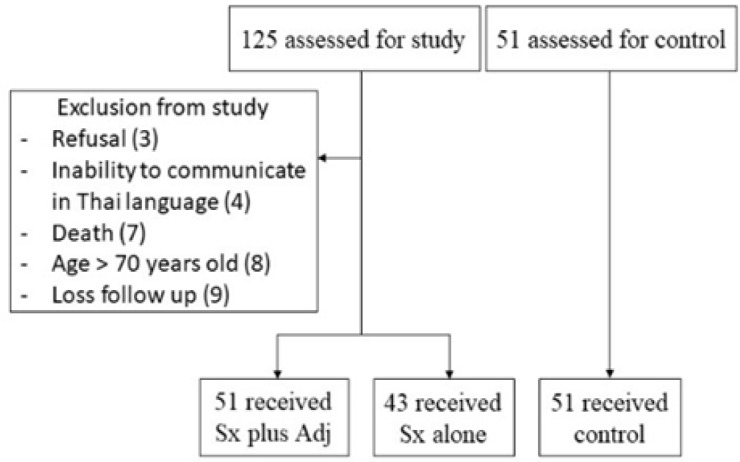
Flow Chart of Study. Sx plus Adj, surgery plus adjuvant radiotherapy with or without chemotherapy; Sx alone, surgery alone; control, healthy women

During the period of the study, 125 participants who were diagnosed with endometrial cancer and underwent surgical staging were enrolled. Thirty-one cases were excluded from the study as presented in Figure 1. A total of 94 cases were registered for the study. Participants who had comparable demographic characteristics and underwent gynecological checkup during the period of enrollment were included into the study as the control group (group C). The study group was divided into surgery with adjuvant therapy (group A) and surgery only (group B). There were 51 and 43 cases in group A and B, respectively.

Participants in group A had higher mean age and lower mean BMI than participants of group B with statistical difference as shown in Table 1. Half of participants in the current study were housewives. One-third of endometrial cases and half of the control group were private company employees and government officers, respectively. However different occupations among participants were not statistically different. Half of participants had monthly income exceeding twenty thousand baht. One third of group A and two thirds of group B/C held a Bachelor’s degree or higher with statistical difference. Half of endometrial cancer cases and three quarters of the control cases currently had partners (couple status). Two thirds of group A/B and one third of group C had underlying diseases namely DM, HT and DLP. Around 80% of participants had no family history of cancer. Participants in group A and B had FIGO staging IA at percentage of 31.4 and 90.7, respectively (p-value< 0.001).

**Figure 2:**
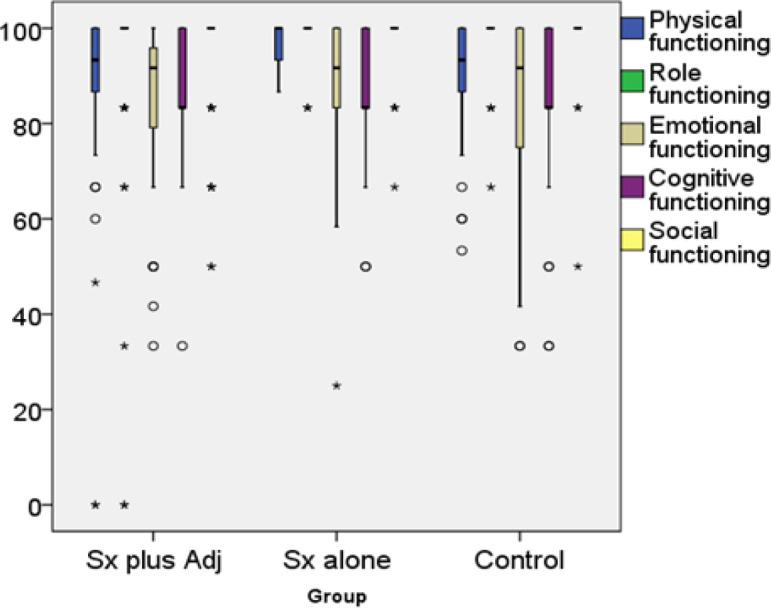
Functional Scores among Surgery Plus Adjuvant Therapy, Surgery Alone and Control. Sx plus Adj, surgery plus adjuvant radiotherapy with or without chemotherapy; Sx alone, surgery alone; control, healthy women

Functional scales from EORTC QLQ C-30 questionnaire among participants in the present study was presented in Figure 2. All three groups had comparable scores in role, emotional and cognitive functioning. In part of physical functioning, cancer cases who underwent surgery only (group B) had better scores than cancer cases who received both surgery and adjuvant treatment (group A) with statistical difference. Participant in group B and C reported significantly better QoL in part of social functioning than group A.

**Figure 3:**
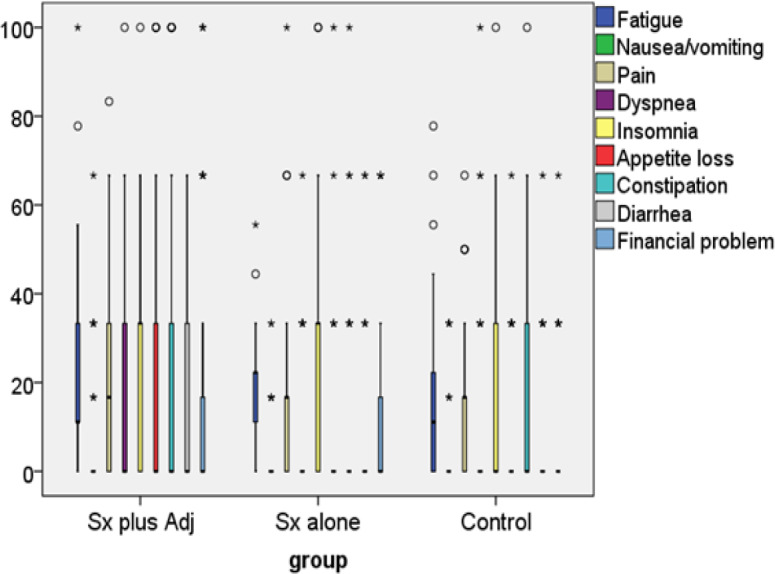
Symptoms Scores among Surgery Plus Adjuvant Therapy, Surgery Alone and Control. Sx plus Adj, surgery plus adjuvant radiotherapy with or without chemotherapy; Sx alone, surgery alone; control, healthy women

Figure 3 presents symptom scales from EORTC QLQ C-30 questionnaire among participants in the current study. Participants in group B had less appetite loss and constipation problems than group A participants with statistical difference. However, participants in group A/C had comparable incidence of constipation problems.

**Figure 4:**
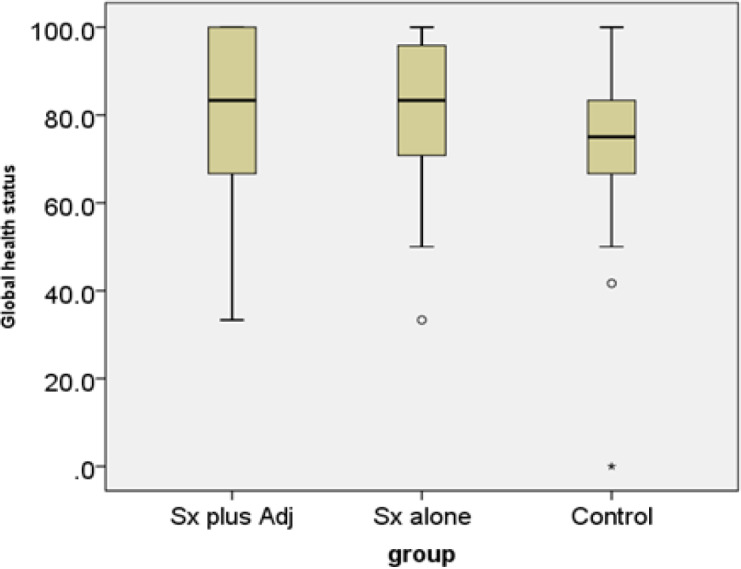
Global Health Status among Surgery Alone, Surgery Plus Adjuvant Therapy and Control. Sx plus Adj, surgery plus adjuvant radiotherapy with or without chemotherapy; Sx alone, surgery alone; control, healthy women

Concerning global health status, the cases in group C had worse global health status than group A/B as shown in Figure 4.

**Table 1:** Demographic Characters among Endometrial Cancer Cases: Surgery Plus Adjuvant Therapy(n=51), Surgery (n=43) and Control (n=51)

	Surgery + Adjuvant*	Surgery*	Control*	*p*-Value
Age(years)**	60.4±8.4	55.8±8.1	47.8±11.1	<0.001
BMI (kg/m²)**	26.1±5.6	28.2±5.6	24.4±3.2	0.002
Occupation				0.125
Housewives	28 (54.9)	19 (44.2)	23 (45.1)	
Agriculture and daily worker	8 (15.7)	5 (11.6)	2 (3.9)	
Private & government officer	15 (29.4)	19 (44.2)	26 (51)	
Income(baht)				0.191
<15,000	24 (47.1)	11 (25.6)	16 (31.4)	
15,000-20,000	5 (9.8)	6 (14)	9 (17.6)	
>20,000	22 (43.1)	26 (60.4)	26 (51)	
Education level				0.002
≤Primary school	27 (41.5)	12 (23.5)	12 (23.5)	
Secondary school	14 (25.5)	10 (15.7)	8 (15.7)	
≥Bachelor	10 (33)	21 (60.8)	31 (60.8)	
Buddhist	50 (98)	42 (97.7)	50 (98)	0.566
Marital status				0.334
Married	29 (57)	23 (53.5)	38 (74.5)	
Single/Divorce/Widow	22 (43)	20 (46.5)	13 (25.5)	
Stay with family	47 (92.2)	42 (97.7)	48 (94.1)	0.501
Healthy insurance				<0.001
Self-payment	2 (3.9)	8 (18.6)	20 (39.2)	
Government	19 (37.3)	22 (51.2)	23 (45.1)	
Social welfare	30 (58.8)	13 (30.2)	8 (15.7)	
Underlying disease	35 (68.6)	27 (62.8)	19 (37.3)	0.003
History of other surgery	26 (51)	26 (60.5)	26 (51)	0.578
Multiparity	38 (74.5)	30 (69.8)	39 (76.5)	0.755
No family history of cancer	38 (74.5)	26 (60.5)	41 (80.4)	0.09
FIGO stage				<0.001
IA	16 (31.4)	39 (90.7%)		
Beyond IA	35 (68.6)	4 (9.3%)		
Histology				<0.001
Endometroid	33 (64.7)	42 (97.7)		
Non endometrioid	18 (35.3)	1 (2.3)		
Grading				<0.001
1	8 (15.7)	18 (41.9)		
≥2	43 (84.3)	25 (58.1)		

*n (%), **mean ± standard deviation (SD); BMI, body mass index; FIGO, The International Federation of Gynecology and Obstetrics; Grade 1, well differentiation; Grade ≥2, moderated and poorly differentiation

## Discussion

Endometrial cancer is the 9th most common female cancer in Thailand (Wilailak et al., 2016). Most endometrial cancer cases were diagnosed at stage I due to vaginal bleeding symptoms which brought patients to see their physicians. Treatment of endometrial cancer was surgical staging (Dowdy et al., 2019). In the early stage of endometrial cancer, surgery alone was sufficient as a proper treatment. On the other hand, early or advanced stage endometrial cancer with adverse risk factors needed further adjuvant treatment after the surgery. Adjuvant treatment consisted of radiotherapy with or without chemotherapy. 

In the present study, half of endometrial cancer cases were diagnosed at stage IA. This finding supported the Thai National Cancer Institute (NCI) uterine cancer report that 10-53 percent of endometrial cancer cases in different regions was diagnosed at stage I (Imsamran et al., 2018). This lead to a high rate of early detection, leading to early surgery. As the only tertiary healthcare provider in the northern suburban Bangkok, patients came from nearby areas with easy access to the service compared to the country’s standard. As a result, mean age of endometrial cancer cases in this finding (58 years old) was considerably lower than that of the normal report in the 6th and 7th decade of life (Dowdy et al., 2019).

Two thirds of our endometrial cancer cases had underlying diseases, i.e., DM, HT and DLP. Women with obesity had 3 to 10 times higher risk of endometrial cancer than average weight population. Relative risk of endometrial cancer in DM patients was 2.8 times higher than non DM counterpart (Dowdy et al., 2019). DLP was a condition closely associated with obesity. An obese woman with DM, HT and DLP with vaginal bleeding was more likely to present with endometrial cancer because of her combined associated risk factors.

*Quality of Life*

Quality of life in endometrial cancer cases was greatly impacted by variable combinations of cancer psychological stress, surgical menopausal symptoms, chemotherapy and radiotherapy side effects (Le et al., 2009). Most cases were found in older women, probably menopausal. The combination of surgery and concurrent chemo-radiation therapy (CCRT) weakened most patients both physically and emotionally. Many found themselves suddenly without partners as their men left after the diagnosis and treatment.

Overall survival rate of endometrial cancer was excellent; 5-year survival in stage I at 87% (Dowdy et al., 2019). The disease was normally found at an early stage with vaginal bleeding. This led to its survivors living for extended periods post treatment. Good quality of life should be considered and advocated for in these women. They normally carry anxiety and fear of possible endometrial cancer recurrent prognosis as well as fighting the burden of their underlying noncommunicable diseases (NCD). Our endometrial cancer survivor population were mainly more than 56 years old and mostly were facing retirement which would greatly alter their finances to a reduced income. Most literature about endometrial cancer survivors and their quality of life came from the European countries (Dobrzycka et al., 2017; Becker et al., 2011; de Boer et al.,2015; Ferrandine et al., 2014; Yavas et al., 2017). Thai studies were needed to understand our population in our socioeconomic and cultural context in order to properly support our patients.

*Functional scales*

Functional scales composed of physical functioning (FPF), role functioning (FRF), emotional functioning (FEF), cognitive functioning (FCF) and social functioning (FSF).

Our surgery with adjuvant therapy endometrial cancer survival group reported the lowest QoL in physical and social functioning domains by a significant margin.

Dobrzycka et al., (2017) reported of Polish study stated that QoL in FEF domain of endometrial cancer survivors (surgery with adjuvant therapy) was worse than those who underwent surgery alone. Those who underwent surgery alone and the control group had similar FEF QoL. The two investigations stated the same finding. However, these findings were different from those of the German, Dutch, and the Japanese literature (Becker et al., 2011; de Boer et al., 2015; Obama et al., 2013). Becker et al., (2011) reported all domains of functioning part among endometrial cancer survivors who underwent surgery with or without adjuvant therapy were comparable. The report of de Boer, Obama and Wang from the Netherland, Japan and China, showed comparable findings with Becker (de Boer et al.,2015; Obama et al., 2013; Wang et al., 2015). Germany, the Netherlands, and Japan are first world economies with solid healthcare and retirement packages. Their survivors supposedly had better peace of mind about their finances, living expenses and medical care coverage. That should explain the no difference QoL in the functioning domain among patients with different treatment. Poland and Thailand both are emerging economies without strong healthcare support from the social structure. It is not surprising for the surgery with adjuvant survivors (the most physically taxing treatment among all groups) to have less QoL about their functional existent.

*Symptom scales*

Symptom scales consisted of fatigue, nausea/vomiting, pain, dyspnea, insomnia, appetite loss, constipation, diarrhea and financial difficulties. In the current study, appetite loss was the major problem among adjuvant treated endometrial cancer survivors. Surprisingly, there was more constipation reports in those who received adjuvant treatment and control cases than those with surgery alone. A possible explanation was that sometimes intact internal genital organs might process a silent gynecological problem such as myoma uteri or endometriosis that could interfere with bowel habits.

Previous literature from Turkey reported that gynecologic cancer survivors (endometrial cancer plus other gynecological cancers) suffered from symptom scales as above mentioned during the first three months of cancer treatment. The symptoms subsided as time went by approaching two years after the treatment and resulted in nonsignificant presentation of the data (Yavas et al., 2017). A Chinese work by Wang and colleagues reported that symptom scales QoL (fatigue, dyspnea, insomnia and constipation) among gynecologic cancer with comorbidity were worse than those without comorbidity (Wang et al., 2015). Obama from Japan reported that symptom scales, i.e., pain, dyspnea and appetite loss of gynecologic cancer survivors (endometrial and cervical cancer) were less than those of premenopausal women (Obama et al., 2013). A previous Thai work by Prasongvej and colleague reported that cervical cancer survivors had better functional QoL than other women with similar characteristics (Prasongvej et al., 2017). However, reports from Poland, Germany and the Netherland yielded similar symptom scales when compared endometrial cancer survivors and those with no cancer (Dobrzycka et al., 2017; Becker et al., 2011; de Boer et al., 2015). Different types of cancer seem to impact patient symptom scales differently. We cannot compare notes between our study and others as they mostly studied gynecological cancer as a group not endometrial cancer alone.

*Global health status*

Our current study found that control cases had lower global health status QoL than that of endometrial cancer survivors. This finding was in lieu with the finding of Prasongvej and colleague’s Thai work in cervical cancer survivors (Prasongvej et al., 2017).

Literature from Poland, Germany, the Netherland, and Italy reported that there was no significant global health status QoL difference between endometrial cancer survivors and their control groups (Dobrzycka et al., 2017; Becker et al., 2011; de Boer et al., 2015; Ferrandine et al., 2014). Chinese and Turkish works reported lower global health status among gynecologic cancer survivors who received wider area of radiation treatment. The wider radiation patients suffered more complications and thus reported lower global health status QoL than their peers (Wang et al.,2015; Yavas et al., 2017). Larger radiation areas usually resulted in radiation cystitis, intra-abdominal adhesion which can cause bowel obstruction with possibility of bursting. It was of no surprise to receive a low global health status QoL scores in wide radiation area survivors. The stark contrast between the global health status QoL finding in Thais’ and European’ works might stem from different beliefs between the cultures. Many Thais believe that cancer is a morbid disease leading to death. Many types of commonly known cancers in Thailand were only found in advance stages, i.e., liver, ovarian and lung cancers. People dread these diseases and think the word “cancer” is associated with “death”. Living after cancer diagnosis and treatment was the big bonus of the survivors’ lives (Prasongvej et al., 2017). It seemed that all treatment complications were not of the survivor’s concerns. Each day of living was a wonderful opportunity. Our participants were relatively of younger age (56+), mostly found their cancers in rather early stage (IA) which resulted in minimum trauma to the treated bodies compared to patients in their sixties as presented in the previous literature. Thai cancer survivors probably thought they had defied death and thus felt gratitude in living and being alive, resulting in high scores in QoL global health domain.

In conclusion, Adjuvant treatment either radiation or chemotherapy had negative impact to QoL in endometrial cancer survivors. Physical, social functioning, appetite loss, and constipation were the major impact factors for endometrial cancer survivors who receive adjuvant treatment. Global health status QoL of all survivors was better than those of the control group because the patients found their diseases in early stage at rather younger ages and were grateful for each day living as survivors. Thorough counseling to endometrial cancer cases who needed adjuvant treatment might play an important role to increase awareness of early complication detection and lower their psychological emotional stress level.

## Conflict of interest

There was no potential conflict of interest relevant to this article.
